# Web Alert: Bacterial cellulose: An annotated selection of World Wide Web sites relevant to the topics in microbial biotechnology

**DOI:** 10.1111/1751-7915.14265

**Published:** 2023-04-25

**Authors:** Lawrence P. Wackett

**Affiliations:** ^1^ Department of Biochemistry, Molecular Biology & Biophysics BioTechnology Institute, University of Minnesota St. Paul Minnesota USA

## Antimicrobial microbial cellulose

### https://www.ncbi.nlm.nih.gov/pmc/articles/PMC8657668/

This paper reviews bacterial cellulose production, with a focus on biocomposites that have antimicrobial and antibiofilm effects.

## Compilation on bacterial cellulose

### https://www.sciencedirect.com/topics/biochemistry‐genetics‐and‐molecular‐biology/bacterial‐cellulose

This page is a compendium of abstracts representing books, chapters and articles related to bacterial cellulose.

## Applications of bacterial cellulose

### https://www.frontiersin.org/articles/10.3389/fbioe.2020.605374/full

This review covers the production of bacterial cellulose with a focus on scale‐up to industrial‐scale production.

## Hybrid materials from bacterial cellulose

### https://www.nature.com/articles/s41428‐021‐00606‐8

Given the commercial interests in bacterial cellulose, both conventional methods and synthetic biology approaches are being used.

## Bacterial cellulose spheroids

### https://www.nature.com/articles/s41467‐021‐25350‐8

This report describes what it calls “engineered living materials” which are fabricated from millimeter‐sized cellulose spheroids from cultures of *Komagataeibacter rhaeticus*.

## Bacterial cellulose applications

### https://www.mdpi.com/2073‐4360/14/6/1080

This paper makes the point that bacterial cellulose is considered to be “generally regarded as safe,” or GRAS, making it useful for food or medical applications. It also has utility for its properties of gel formation, emulsifying properties and water retention.

## Mechanism of cellulose synthase activation

### https://www.nature.com/articles/nsmb.2803

This is one of the early reports describing the stimulation of bacterial cellulose synthesis by the signaling molecule cyclic di‐GMP.

## Cellulose synthase BcsB structure

### https://www.rcsb.org/structure/7LBY

This site contains the coordinates for BcsB that comprises most of the approximately one megadalton Bcs cellulose synthase complex.

## Bacterial cellulose synthase in action

### https://www.rcsb.org/structure/5EJ1

This structure and the accompanying paper describe snapshots along the pathway of cellulose synthesis and its translocation to the exterior of the cell. The protein system was expressed in *Escherichia coli* but originated from *Cereibacter sphaeroides*.

## Cellulose synthesis by *Komagataeibacter hansenii*

### https://www.rcsb.org/structure/5EJ1

*Komagataeibacter hansenii* ATCC 53,582 is important commercially because it produces cellulose in relatively high yield. This study looked into some of the enzyme complexes in cellulose biosynthesis and the networks involved in its regulation.

